# Nurses and midwives professional support increases with improved attitudes - design and effects of a longitudinal randomized controlled process-oriented intervention

**DOI:** 10.1186/s12884-015-0712-z

**Published:** 2015-10-26

**Authors:** Anette C Ekström, Stina Thorstensson

**Affiliations:** School of health and education, University of Skövde, Post box 408, S 541 28 Skövde, Sweden

**Keywords:** Childbirth, Childbearing, Breastfeeding, Parental support, Interaction, Pedagogy, Professional support, Professional attitudes

## Abstract

**Background:**

Becoming parents for the first time is challenging. Mothers need both social and professional support to handle these challenges. Professionals’ attitudes affect quality of care and support. So to improve professional support, an intervention consisting of a process-oriented training was performed. Due to the positive results of the intervention there is a need to illuminate the methodological approach further. The overall aim was therefore to describe a methodological approach to improve and evaluate health care professionals’ attitudes toward breastfeeding and parental support in order to improve quality of care in childbearing.

**Methods:**

This study was a longitudinal randomized control intervention study, in which groups of mothers received care in childbearing from midwives and child health nurses. These health professionals had gone through a process-oriented training, or not. In order to improve attitudes of health professionals the training was based on evidence, practical skills and reflective processes (both private and professional experiences) in relation to breastfeeding and parental support. Included in the longitudinal study were health professionals from five intervention municipalities *n* = 36 and health professionals from five control municipalities *n* = 45. All mothers who fulfilled the inclusion criteria were consecutively identified from the hospital register and asked to participate in the study. Mothers who accepted to participate were included in the interventions group (*n* = 206) or control groups (*n* = 162, *n* = 172 respectively) based on which municipality they belonged to.

**Results:**

The results of the process-oriented training improved the professionals’ attitudes toward breastfeeding and parental support. These improved attitudes in health professionals increased intervention-group mother’s satisfaction with professional and social support. Intervention-group mother’s relation to and feelings for their baby as well as breastfeeding was also improved.

**Conclusion:**

These results stress the importance of professionals’ attitude in quality of care during childbearing, as well as pointing to the possibility to improve professionals’ attitudes with a process-oriented training.

**Trial registration:**

Australian New Zealand Clinical Trials Registry (ANZCTR), trial registration: ACTRN12611000354987.

## Background

To become parents for the first time is a major change of life event [1, 2]. It involves the physiological endeavour of pregnancy, birth and breast-feeding, but also changes in social lives [3, 4]. To handle these challenges mothers need both social and professional support [5, 6]. Support has been described as an interactive process that affects wellbeing and health of the individual [7]. Social support is offered within individuals own network and then working relationships and trust is required for support to be effective. Professional support, however, is directly available but limited by professional domain and knowledge, for example childbearing and midwifery [8]. So it is important to differentiate between social and professional support in research [9]. Care interventions with professional support should also aim to strengthen social support [10]. In childbearing, professional support from nurses and midwives is important for parents’ childbirth experience [11–14]; for mother infant interaction [15–17] and for breastfeeding [18]. Health professionals’ personal attitudes affect quality of care and support as well as their perception of the support they offer. This will affect professionals ability to meet patients’ individual needs, however more research is needed [19]. So to achieve improved quality of care, a change of attitude among health care professionals is needed. To change attitudes an approach with both fact and reflection has been described [20] and reflection is essential for professionals’ skills development [20, 21]. Therefore an intervention with a process-oriented training including evidence based lectures and reflection was performed to change health professionals’ attitudes toward breastfeeding and parental support. This intervention resulted in health professionals attitudes becoming more facilitating and less regulating [22], mothers experienced improved professional support, improved self-reported relation to and feelings for the baby and breastfeeding [15, 17, 23–28]. Due to these positive results there is a need to illuminate the methodological approach further. The overall aim is therefore to describe a methodological approach to improve and evaluate health care professionals’ attitudes toward breastfeeding and parental support in order to improve quality of care in childbearing.

## Methods

### Design

**Fig. 1:**
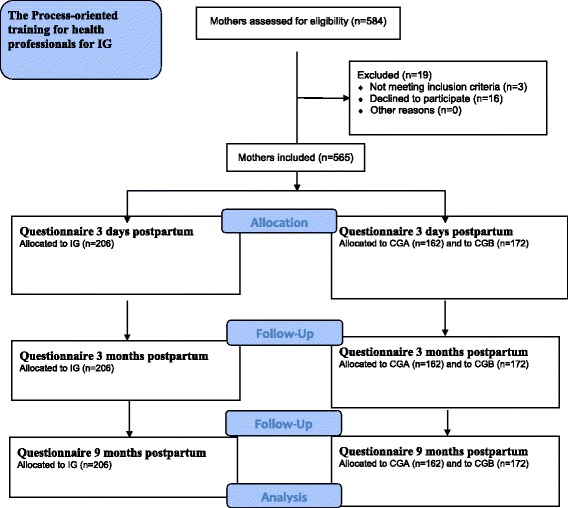
Flow diagram of how mothers enrolled in the Intervention group (IG), Control Group A (CGA) and Control Group B (CGB)

This was a longitudinal randomized control intervention study, in which groups of mothers received care in childbearing from antenatal midwives and child health nurses (here referred to as health professionals). The health professionals had gone through a process-oriented training in breastfeeding and parental support, or not. The group of health professionals that had not gone through the process-oriented training could be considered as the standard care group. The study design follows the Consort recommendations and it is registered in Australian New Zealand Clinical Trials Registry (ANZCTR), trial registration: ACTRN12611000354987. The study design is presented in Fig. 1.

### Setting

The study was performed in a county in the southwest of Sweden, with 280,000 inhabitants, consisting of 13 municipalities with antenatal and child health centres and comprised of urban, suburban, and rural districts. Approximately 2500 births occurred annually at the two hospitals at this time period. Mothers and their partner would meet a midwife for pregnancy medical check-ups, parental education and support approximately eight to eleven times during pregnancy. Almost all mothers gave birth in hospital, and care in hospital was provided by midwives who were not previously known to the mothers. The average length of hospital stay was between six hours to seven days, and a child-health nurse made a home visit seven to ten days after the birth, and remained in contact until the baby was old enough to start school at six years of age. Visits to the child-health nurse included medical check-ups of the child and information/support in breast-feeding and parenting. Results from a mapping study showed mothers lacked information and support in breast-feeding and parenting [29].

### Intervention

This intervention has two phases; the first phase was the process-oriented training to improve health professionals’ attitudes and the second phase was evaluating whether health professionals improved attitudes had effect on breastfeeding, feeling and relation to the baby and mothers’ experiences of support.

#### Phase 1: The process-oriented training for midwives and child health nurses (health professionals) in breastfeeding and parental support

##### First part

Allocation of municipalities to intervention or control groups. Based on the findings of a baseline study [29, 30], the ten largest municipalities in the selected area were paired according to their sizes and duration of breastfeeding. For each pair of municipalities, one was then randomly designated to the five-municipality intervention group and one to the five-municipality control group. Then antenatal midwives and child health nurses were allocated to intervention or control depending on whether their work site had been selected as an intervention municipality or as a control municipality. In total 116 health professionals were asked to participate in the study [15, 17, 22–28]. A Breastfeeding Attitude Instrument was developed to measure breastfeeding attitudes in health care professionals (*n* = 168), Four attitude dimensions were identified by factor analysis [31].

##### Second part

**Fig. 2:**
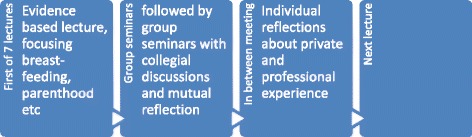
The process of the training

Thus, all antenatal midwives and child health nurses working at the randomised intervention municipalities accepted to participate in the study (*n* = 81). A process-oriented training [20] in breastfeeding and parental support was conducted for midwives and child health nurses (together referred to as ‘health professionals’ for the remainder of this report) from the intervention municipalities. Figure 1 describes flow diagram of included mothers, Fig. 2 demonstrates the process of the training and Appendix 1 describes the content of the process-oriented program. Health professionals’ attitudes were evaluated with the breastfeeding attitude instrument, before, immediately after and one year after the process-oriented training [22].

#### Phase 2: Evaluation of effects for mothers of the process-oriented training for health professionals

Mothers who were included in the study had either been cared for by health professionals in one of the five intervention municipalities or by health professionals in one of the five control municipalities. None of the mothers knew whether their antenatal midwife and child health nurse had gone through the process-oriented training (intervention groups) or not (control groups). During their stays at the delivery and maternity ward at the hospital, all the mothers met midwives who had not participated in the process-oriented training.

##### Inclusion criteria

Mothers who were Swedish-speaking, healthy first-time mothers and who gave birth to single, healthy full-term babies delivered spontaneously, by vacuum extraction, or by Caesarean section were eligible.

##### Exclusion criteria

Mothers who were first-time mothers and who had given birth to babies with malformations or life-threatening diseases, for example life-threatening illness such as very severe asphyxia, were excluded.

All mothers who fulfilled the inclusion criteria and had been cared for at the antenatal and child health clinics in the municipalities selected for this study were consecutively identified from the hospital register and asked to participate in the study (*n* = 584) (Fig. 1). Of those, 480 gave their informed consent to participate, which translates to a response rate of 82 % (Table 1). Before the process-oriented training commenced and any effects of the intervention could be measured, data were collected for a baseline group called Control Group A (CGA, n = 162). Data for Control Group B (CGB, n = 172) and Intervention Group (IG, n = 206) were collected simultaneously. This design allowed detection of changes over time and any spill over effects of the intervention. The same five municipalities provided the sample population for CGA and CGB. Sociodemographic and obstetric data for included mothers is presented in Table 2.

**Table 1:** Response rate for all groups at 3 days, 3 months, and 9 months postpartum

	IG *n* = 206		CGA *n* = 162		CGB *n* = 172		Total *n* = 540	*p*
3 days postpartum, n (%)	172	(84 %)	148	(91 %)	160	(93 %)	480 (89 %)	n.s
3 months postpartum, n (%)	145	(70 %)	126	(78 %)	132	(77 %)	403 (74 %)	n.s
9 months postpartum, n (%)	131	(64 %)	116	(72 %)	125	(73 %)	372 (69 %)	n.s

The intervention group (IG), Control Group A (CGA) and Control Group B (CGB)

**Table 2:** Sociodemographic and obstetric data for mothers in all groups at three days after

	IG 172		CGA 148		CGB 160	
Age in years (m and SD)	26.6	(4.5)	27.2	(4.6)	27.0	(5.0)
Gestational weeks (m and SD)	40.4	(1.4)	40.5	(1.4)	40.4	(1.4)
Education						
Compulsory school (%)	6	(3 %)	5	(3 %)	3	(2 %)
High school (%)	77	(37 %)	73	(45 %)	71	(41 %)
University (%)	74	(36 %)	55	(34 %)	62	(36 %)
Other (%)	14	(7 %)	15	(9 %)	21	(12 %)
Missing	35	(17 %)	14	(9 %)	15	(9 %)
Marital status						
Cohabitation (3 days postpartum)	125	(61 %)	102	(63 %)	118	(69 %)
Married	42	(20 %)	43	(27 %)	38	(22 %)
Single	3	(1.5 %)	2	(1 %)	2	(1 %)
Other	1	(0.5 %)	3	(2 %)	2	(1 %)
Missing	35	(17 %)	12	(7 %)	12	(7 %)
Obstetric data						
Vaginal delivery (%)	146	(70 %)	120	(74 %)	129	(75 %)
Caesarean section (%)	32	(16 %)	22	(14 %)	31	(18 %)
Vacuum extraction/forceps (%)	28	(14 %)	20	(12 %)	12	(7 %)

The intervention group (IG), Control Group A (CGA) and Control Group B (CGB)

### Evaluation of health professionals improved attitudes effect for mothers

Three questionnaires were developed about professional (MoPPS-scale) and social support, mothers’ self-reported feelings for and relation to the baby (MIRF-scale) and breastfeeding. Maternity staff members distributed the first questionnaire to the mothers three days after birth. Follow-up questionnaires were posted to the mothers three months and nine months after birth (Fig. 1). Obstetric and demographic data were collected from birth records, and socio-demographic background data were collected with the first questionnaire (Table 2). The three questionnaires developed for this study were pilot-tested by 20 mothers for acceptability and face validity. In addition, an expert group of health professionals was consulted to establish content validity. A few minor corrections to the wording were made before the data collection began.

### Statistics

For the statistical analyses of the data, we used the Statistical Package for the Social Sciences (SPSS) version 13, 14 & 15. Central measurements were presented as a mean (M) and dispersion by standard deviation (SD). To test the differences between the groups, one-way ANOVAs and Tukey’s HSD-test for post hoc comparisons were performed. Chi-square tests were performed on category data. Pearson’s rank correlation was used to relate data on the ordinal level. In order to reduce the amount of data tested all items representing the mothers’ relation to and feelings for the baby were entered into a principal component factor analysis using varimax rotation. A forward multiple linear regression analysis was performed to examine the impact of the obstetrical variables [32]. In one of the studies [17] Cohen’s guidelines were used to calculate the effect sizes in order to interpret clinical change, and the effect was defined as small (> 0.20), moderate (> 0.50), or strong (> 0.80) [17, 33]. *P*-values ≤ 0.05 were considered significant [32].

### Ethical considerations

We obtained ethical approval from the Ethical Review Board of Gothenburg (EPN) before any data collection began, register number L 188–99, L 005–98 and 405–09. The head of the organization was given information about the study and gave us access to undertake this study. The participating first-time mothers were given written and oral information about the study, as well as information explaining that their participation was voluntary and that they could withdraw from the study at any time without having to provide a reason and without their care being affected.

## Results

Effects of this intervention has been published in scientific journal and a book chapter (Table 3).

**Table 3:** Overview of publications from the Intervention

Ref nr	Authors	Title	Journal	Year of publ
31	A.Ekström, A.Matthiesen, A.Widström, E.Nissen	Breastfeeding attitudes among counselling health professionals. Development of an instrument to describe breastfeeding attitudes.	Scandinavian Journal of Public Health, 33(5), 353–359.	2005
22	A.Ekström, A.Matthiesen, A.Widström, E.Nissen	Process-oriented Training in Breastfeeding Alters Attitudes to Breastfeeding in Health Professionals.	Scand. J of Public Health, 33(6), 424–431.	2005
28	A.Ekström, A.Matthiesen, A.Widström, E.Nissen	Does Continuity of Care by Well‐Trained Breastfeeding Counselors Improve a Mother’s Perception of Support?	Birth, 33(2), 123-130	2006
15	A.Ekström, E.Nissen	A mothers’s feelings for her baby are strenghten by excellent breastfeeding counseling and continuity of care.	Peadiatrics, 118(2), e309-e314.	2006
27	A.Ekström, E.Nissen	Process-Oriented Training in Breastfeeding Attitudes and Continuity of Care Improve Mothers Perception of Support.	Health Education Research Trends. Nova Publishers. NY.	2007
25	A.Ekström, K.Guttke, M.Lenz, E.Hertfelt Wahn	Long term effects of professional breastfeeding support - An intervention.	International Journal of Nursing and Midwifery, Vol. 3(8), pp. 109–117.	2011
26	A.Ekström, E.Kylberg E.Nissen,	A process-oriented breastfeeding training for health professionals to promote breastfeeding.	Breastfeeding Medicine. 7(2), 85–92.	2012
17	S.Thorstensson, E.Nissen, A.Ekström	Professional support in pregnancy influence maternal relation to and feelings for the baby after Cesarean birth; an intervention study.	Journal of Nursing & Care 1:112.	2012
23	I.Blixt, L.B.Mårtensson, A.Ekström	Process-oriented training in breast feeding for health professionals decreases breastfeeding challenges.	International Breastfeeding Journal, 9:15	2014
24	A.Ekström, H.Abrahamsson, R-M.Eriksson L.B.Mårtensson,	Women’s use of nipple shields - its influences on breastfeeding duration after a process oriented education for health professionals.	Breastfeeding Medicine, 9(9), 458-466	2014

### Professionals’ breastfeeding attitudes

Effects of the process oriented training (Fig. 2 and Appendix 1) in relation to professionals’ breastfeeding attitudes was measured with the breastfeeding attitude instrument [22] which can be used for accurate assessment of health professionals’ breastfeeding attitudes. By means of factor analysis four factors were identified: the “Regulating” factor focused on regulating mothers’ breastfeeding management, the “Facilitating” factor focused on making it easy for mothers to manage their breastfeeding, the “Disempowering” factor focused on giving advice, disregarding the needs of the woman being counselled and the “Breastfeeding antipathy” factor focused on insufficient, basic, breastfeeding knowledge and aversive reactions to breastfeeding [22]. After one year, health professionals in the intervention group reduced their scores on the regulating scale (*p* <0.001) and increased their scores on the facilitating scale [27] when compared with control group health professionals.

### Improved professional attitudes effect for mothers

#### Mother’s perception of professional support

Overall, mothers in the IG reported more a positive perception of professional support, than mothers in the CG [28]. In addition, IG mothers with a caesarean birth reported more positive on professional support during pregnancy than CG mothers [17]. IG mothers also reported a more positive perception of professional support from staff at the labor and maternity ward than CG mothers [25], despite the fact that these professionals were not included in the process-oriented training. IG mothers reported improved professional support in preparation for the parental role and their partners were more active in parental support groups than CG mothers partners were. IG mothers also reported stronger social support from other parents than CG mothers did [28].

#### Mother’s feelings and relation to the baby

As a result of improved professional support IG mothers reported stronger for feelings and relation to the baby than CG mothers did [15]. In addition, IG mothers with a caesarean birth also reported stronger for feelings and relation to the baby than CG mothers with a caesarean birth did [17].

#### Positive influence in breast-feeding

IG mothers reported improved breastfeeding support leading to earlier initiation, higher frequency of breast-feeding the first 24 h [25], less breast-milk substitute in the first week of life (without medical reasons) and introduction of breast milk substitutes after discharge from the hospital, were delayed in relation to CG group mothers [26]. In addition, IG mothers reported less breast-feeding challenges such as; experiencing insufficiency in breast-milk, the first three months after birth [23], despite the use of nipple shields or not [24]. IG mothers also reported longer breastfeeding duration than CG mothers did [15, 23–28].

## Discussion

This article describes a methodological approach, a process-oriented training, which improved midwives and child health nurses attitudes toward breastfeeding and parental support. Improved attitudes in health professionals lead to mothers being more satisfied with professional support. In addition the improved professional support increased mothers feelings and relation to the baby, earlier initiation and higher frequency of breast-feeding the first 24 h, less breast-milk substitute (without medical reasons), less breast-feeding challenge (despite the use of nipple shields) and as a result of these, longer breastfeeding duration.

These improved attitudes in health professionals tended to be stable over time and IG mothers reported more satisfaction with emotional and informative professional support during the first nine months after birth than CG mothers. In line with other research, quality of care is improved by enhancing health professionals’ attitudes [34]. since health professionals attitudes influence their ability to meet individual needs of patients [21, 35]. In order to improve attitudes both fact and reflection is needed [20] which is essential for skills development in professional education [20, 21]. This is in line with our result that a process-oriented training which integrated facts with reflections on both professional and personal level lead to improved professional attitudes.

In evaluation of the process-oriented training, health professionals described that they did not have adequate time available to pursue the education during working hours. They described that despite decision making heads expressed an interest in the training the health professionals experienced that they did not have reasonable conditions to pursue the course (unpublished data). This points to lack of understanding among heads of organizations that competent health professionals require reasonable conditions to develop good quality in health care [36]. Evidence based health care demand possibilities for health professionals to develop their abilities and knowledge during working hours. Heads of health care should not assume that midwives and nurses shall dedicate their free time for professional development.

IG mothers reported a more positive perception of professional support during childbearing as a result of the intervention. However, our results also highlight the fact that IG mothers reported more positive support from the professionals in the delivery ward and the maternity ward, despite the fact that these health professionals were not involved in the process-oriented training. Social and professional support is affected by earlier experiences of receiving support [7, 37], and positive memories of a situation influence a friendly approach in a similar situation [38]. It is possible that mothers who have had a more positive experience of support during antenatal care are more likely to have a positive perception of support from other health professionals as well. Mothers in the IG also reported improved professional support in preparation for the parental role and their partners were more active in parental support groups compared to CG mothers. This result is in line with other researchers that highlights professional support as important for parent’s childbirth experience [11–14].

In addition results from our studies showed that improved professional support increased IG mother’s feeling and relation to the baby, including IG mothers with caesarean birth who are particularly vulnerable. Mothers with caesarean birth are more likely to suffer from psychosocial health problems after childbirth [39–41], and they seem more self-oriented and less self-confident when caring for the baby two months after birth [40], which may negatively influence mother-infant interaction [42, 43]. These results point to the importance of improving professional attitudes to improve support and quality of care for mothers and families in childbirth.

As a result of the process-oriented training mothers in the IG reported earlier initiation, higher frequency of breast-feeding the first 24 h [25], less breast-milk substitute (without medical reasons) [26], less breast-feeding challenge despite the use of nipple shields and as a result of these increased breastfeeding duration [24] compared to CG mothers. Our result is in line with other research showing that when health professionals receive breastfeeding education based on WHO guidelines, they feel more secure and experience an increased ability to support mothers with coherent, evidence-based counselling [44]. Other research shows that when caregivers have communication skills, their ability to empathize and find individual solutions increases, which reduces the risk that mothers perceive the advice as contradictory [45–47]. Mothers need to receive realistic, consistent and evidence-based information on breastfeeding [48] and a combination of group and individual counselling is more efficient for breast-feeding success [49]. Mothers with higher knowledge of breastfeeding also have more confidence in their ability to breastfeed [50].These results may affect mother’s ability to manage their breastfeeding problems better, depending on whether the breastfeeding counselling was more suited to their needs and their life situation.

This longitudinal intervention method with two control groups (CGA data was collected before any effects of the intervention could be measured) was selected as being suitable for the study. This is a design suggested to measure possible spillover effects [51]. More differences were found when the IG was compared with the CGA than when the IG was compared with the CGB (where data were collected simultaneously with the IG). These results show that changes also take place among controls when an intervention is being rolled out. In the professional network of midwives and child health nurses, knowledge and information are shared, which easily leads to spillover effects between intervention and control professionals. These results thus demonstrate the value of using a historic control group, which will provide a baseline against which to evaluate the spillover effect. Strength of the study was that the participating mothers did not know whether they encountered health professionals who had undergone the process-oriented training or not.

## Conclusion

A methodological approach as a process-oriented training for midwives and child health nurses, improved their attitudes toward breastfeeding and parental support. In addition these improved attitudes increased IG mother’s satisfaction with professional and social support as well as their relation to and feeling for the baby and breastfeeding. These results stress the importance of professionals’ attitude in quality of care during childbearing, as well as pointing to the possibility to improve professionals’ attitudes with a process-oriented training.

## Appendix 1

The process-oriented training for healthcare professionals.

Definition of the process-oriented training: In order to improve attitudes of health professionals the training was based on evidence, practical skills and reflective processes (both private and professional experiences) in relation to breastfeeding and parental support. To ensure that the healthcare professionals would be ready to meet the demands of their profession they were trained in problem solving, self-reflection, and decision-making (in terms of competence), and their personal qualifications evaluated (Jerlock, Falk & Severinsson (2003)).

The process-oriented training involved seven days of lectures and its main theme focused on the participants’ breastfeeding experiences (both private and professional), breastfeeding attitudes, breastfeeding counselling, and collaboration and communication between antenatal centers and child health centers in line with WHO recommendations*. Midwives and postnatal nurses were asked to reflect on different areas relating to breastfeeding support. The training supervisors were chosen to strengthen the process between the healthcare centers and the hospital wards. Both the literature and the lecturers raised parenthood, breast-feeding, relations, attachment, the baby’s ability as well as complications around childbearing as well as a common parental education with both midwives and postnatal nurses included. It was the same parental education groups during pregnancy and the babies first year of life.

The healthcare professionals chose the following topics in order to offer an individualized professional support during childbirth:

- ▪ reflections on how breastfeeding occurred in the participants’ lives, both private, in education and professional life,
- ▪ the woman’s physical and mental adaptation to the breastfeeding situation,
- ▪ the infant’s own power, and senses, both as unborn and as newborn,
- ▪ parents relations and parental leave, in a gender and cultural perspective,
- ▪ protect, promote, and support breastfeeding and family health,
- ▪ parent-infant interaction and
- ▪ in history of a global and organizational perspective.

Different themes were treated in each group, these were then reported in the cross-groups. A written summary was made so that everyone could take part of everyone’s work. The entire course ended with the professionals at every municipality did their own suggestions on parental education together. Parental education would be implemented by the professionals together during the pregnancy and baby’s first year.

Jerlock M, Falk K, Severinsson E. 2003 Academic nursing education guidelines: Tools for bridging the gap between theory, research and practice. Nurs Health Sci 5: 219–228.
